# Experimental evolution of active Brownian grains driven by quantum effects in superfluid helium

**DOI:** 10.1038/s41598-022-09523-z

**Published:** 2022-04-12

**Authors:** Oleg F. Petrov, Roman E. Boltnev, Mikhail M. Vasiliev

**Affiliations:** Joint Institute for High Temperatures, Russian Academy of Sciences, Moscow, 125412 Russia

**Keywords:** Quantum fluids and solids, Colloids, Thermodynamics

## Abstract

Complex structures, consisting of a large number of interacting subsystems, have the ability to self-organize and evolve, when the scattering of energy coming from the outside ensures the maintenance of stationary ordered structures with an entropy less than the equilibrium entropy. One of the fundamental problems here is the role of quantum phenomena in the evolution of macroscopic objects. We provide experimental evidence for the active Brownian motion and evolution of structures driven by quantum effects for micron-sized grains levitating in superfluid helium. The active Brownian motion of grains was induced by quantum turbulence during the absorption of laser irradiation by grains. The intensity of Brownian motion associated with quantum vortices increased by 6–7 orders of magnitude compared to the values from the Einstein formula. We observed the grain structures in a state far from thermodynamic equilibrium and their evolution to more complex organized structures with lower entropy due to the quantum mechanism of exceedingly high entropy loss in superfluid helium.

## Introduction

The observation of processes in nature and social phenomena shows that many complex structures, consisting of a large number of interacting subsystems, under certain conditions have the ability to self-organize and evolve. The structures are called dissipative, provided that the scattering of energy coming from outside provides a stationary ordered structure with an entropy less than the equilibrium one. The dissipative structures are capable of self-organization and evolution while increasing the flow of entropy into the environment^1–4^.

Self-organization is considered as an elementary process of evolution consisting of an unlimited sequence of self-organization processes and leading to the formation of more complex structures of the entire system. Along with dissipative self-organization, there are other types, such as conservative self-organization (formation of crystal structures, biopolymers, etc.) and dispersive self-organization (formation of soliton structures)^1,2^. Note that living systems are distinguished among dissipative structures^1–4^. The basic feature of such systems is spontaneous evolution, which is the development of more complex forms due to a sequence of self-organization processes^1,2,4,5^. The fundamental problem here is the thermodynamics of prebiological evolution, when a prebiological system can evolve through a whole sequence of transitions leading to a hierarchy of increasingly complex and organized states. These transitions can occur only in nonlinear systems far from thermodynamic equilibrium^4,6^.

Here, entropy is a key physical quantity when describing self-organization; it serves as a measure of disorder so that a decrease in entropy in the system leads to self-organization. The entropy of the system can decrease if the system exports entropy so that the export per unit of time exceeds the corresponding production of entropy in the system. This process requires an entropy pump. To drive the pump, as for driving any machine, a consumable free energy or free enthalpy is required, which the pump can take from external or internal sources^1,2^.

In recent years, active Brownian motion has attracted great interest in biology, physics, sociology, material science, and epidemiology. While passive Brownian grains are in thermal equilibrium with their environment, active Brownian grains are able to absorb energy from their environment and turn it into their kinetic energy that displaces them from thermodynamic equilibrium^7^. Thus, the systems of active Brownian grains can be considered as open systems, and the structures of grains themselves are systems far from thermodynamic equilibrium.

Active Brownian grains can be charged grains, suspended in plasma or levitated in a cryogenic liquid (cryogenic colloid), the kinetic motion of which is induced by laser radiation^8,9^. In this case, the state of grains is maintained by the free energy of radiation.

One of the fundamental problems in modern physics is the role of quantum effects at the macroscopic scale (in the macroscopic world)^10^ and, in particular, in the evolution of macroscopic structures of matter. Thus, a necessary condition for the evolution of dissipative structures is the outflow (export) of entropy into the environment. Such export can be created, for example, by heat transfer due to radiation or by the mechanism of heat transfer in the environment. During heat transfer in condensed matter, an exceedingly high rate of heat (and entropy) loss is achieved in a quantum liquid (superfluid helium). Therefore, macroscopic phenomenon such as the evolution of colloidal systems in superfluid helium can be associated with quantum effects^11^. The mechanism of activity of Brownian grains in superfluid helium, when they absorb radiation energy and can be heated, can also have a quantum nature associated with the formation of quantum turbulence near the heated grain surface^12,13^.

In this paper, we present the results of the experimental evidence of the evolution of the spatial structures of highly charged (up to 10^5^ *e*) micron-sized grains levitating in a static magnetic trap^8,14–19^ in superfluid helium ^4^He at temperatures *T*_*He*_ = 1.7–2.18 K. The levitation of the grains made with high-temperature superconducting ceramics^20^ is based on the well-known Meissner effect of expulsion of the magnetic field from the volume of the superconducting phase^21,22^. Such systems can be considered colloidal structures in a cryogenic liquid (cryogenic colloid)^8^.

We observed the formation and evolution of a complex structure maintained far from equilibrium by the energy of laser radiation. The structure consisted of a cloud of single grains and spatially oriented chains with strong Coulomb and magnetic intergrain interactions. The absorption of light and heat release on the surface of grains at a certain laser power can initiate the appearance of quantum vortices in superfluid helium and the generation of quantum turbulence^12,13^. It is revealed that an increase in the power density of laser radiation increases the kinetic energy of the motion of grains and their diffusion coefficient by many orders of magnitude in comparison with the equilibrium values at the temperature of superfluid helium.

The study of the spatial structures of grains under the action of laser radiation has also been carried out in low-pressure gas discharges, both at room temperature and at temperatures of superfluid helium^23,24^.

Surprisingly, another observed effect is the increase in the number and length of grain chains with the increase in the power density of laser radiation. It should be noted that an increase in the kinetic energy of grains in the chain structures in the low-pressure gas discharges leads to the destruction of chain structures and the transition of the system to a less ordered state^24,25^.

## Experiments

To study experimentally the strongly interacting Coulomb structures, a levitation of grains confined in an inhomogeneous stationary magnetic field is commonly employed^14,15^. Graphite, as a material with a great value of specific magnetic susceptibility, is of frequent use in experiments on grain confinement in nonuniform magnetic fields^16,17^. Under the conditions of laboratory experiments, at magnetic fields of ~ 2 T/mm, the authors obtained a cluster consisting of only a few graphite grains with sizes of several hundred microns. In experiments on board the International Space Station under microgravity conditions, the formation of an extended cluster of several thousand charged diamagnetic grains, tens and hundreds of microns in size, was observed^18^. The method of magnetic self-assembly of diamagnetic spheroids was also used by the authors to print living tissues^19^.

**Figure 1:**
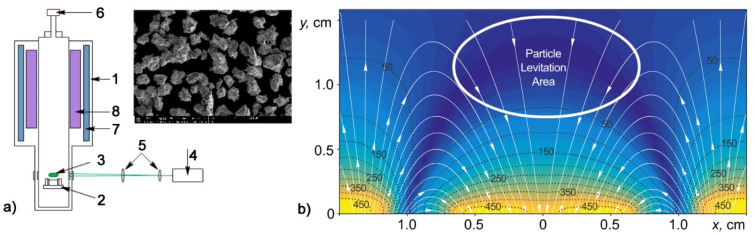
Experimental setup and magnetic trap: (**a**) Scheme of the experimental setup: 1—optical cryostat; 2—platform for the magnetic trap; 3—captured grains in the trap; 4—laser for illumination of YBa_2_Cu_3_O_7_ grains in a trap; 5—lenses; 6—vacuum gauge; 7—bath with liquid nitrogen; 8—bath with liquid helium; (**b**) The distribution of the magnetic field (mT) in the trap for confining YBa_2_Cu_3_O_7_ grains: the thin white lines correspond to the lines of force, and the black dashed lines correspond to the equipotential surfaces of the magnetic field with the denoted values (mT). Inset: Electron microscope image of YBa_2_Cu_3_O_7_ grains.

We used a levitation of micron-sized grains confined in an inhomogeneous stationary magnetic field with induction ${B}_{m}$. Stable levitation of diamagnetic bodies occurs in the region of a local minimum of the magnetic field; however, under conditions of gravity, magnetic fields can be used without a local minimum (see Fig. 1). In this case, the force ${{F}_{d}=(\chi }_{p}{V}_{p}/{\mu }_{o})\nabla {(B}_{m}^{2})$ is balanced by gravity (here, ${\mu }_{o}$ vacuum permeability, ${\chi }_{p}$ magnetic susceptibility of grain material, and ${V}_{p}$ grain volume^8,16–18^).

### Experimental design

The main part of the experimental setup is a Janis SVT-200 optical helium cryostat with an operating temperature range of 1.5–273 K (see Fig. 1a). A special insert is placed in the central vertical channel of the cryostat, which is 62 mm in diameter. At the lower part of the insert, there is a platform for installing magnets that form a magnetic trap. The platform is fixed at the level of the cryostat optical windows. A semiconductor thermometer TPK-1.5/60–22 is attached to the platform. The thermometer is operated with a temperature controller (Lake Shore 335).

In our work, an assembly of a pair of permanent axial magnets made of NdFeB was used as a magnetic trap. The outer ring, in the form of a ring with outer and inner diameters of 35 and 20 mm, respectively, had a height of 8 mm and a residual magnetic induction of 1.43 T. The inner cylinder, in the form of a cylinder with a diameter of 15 mm and a height of 5 mm, had a residual magnetic induction of 1.46 T. The induction vectors of the magnets in the assembly were directed in opposite directions. To maintain the mutual orientation of the magnets, an insert in the form of a ring made of a nonmagnetic material, stainless steel, was installed in the gap between them. The configuration accuracy of the magnets was ± 0.1 mm. Grains made of the high-temperature superconductor YBa_2_Cu_3_O_7_ with a critical temperature of 93 K were injected into a trap, and the grain sizes ranged from 30 to 60 μm (see subplot in Fig. 1a). An injector was located 6 cm above the magnets. The injector holder, injector body and platform for magnets are made of a diamagnetic material—polyamide-6. When injected at temperatures higher than the critical temperature, grains fell onto magnets and were charged up to potential *φ* applied to magnets. The transition of YBa_2_Cu_3_O_7_ grains to the superconducting state upon cooling led to the formation of a cloud captured in a magnetic trap (see in Fig. 1b the magnetic field distribution of the trap was measured with a digital magnetometer Aktakom ATE-8702) and to the appearance of spatial structures of grains in it.

The main elements of the diagnostic complex of the experimental setup were a high-speed digital video camera IDT X-Stream, a solid-state laser with a wavelength of λ = 532 nm with an output power up to 1.0 W and a personal computer with a package of specialized programs for grain detection, video recording, and video data processing. Visualization of grains levitating in a magnetic trap was carried out by illuminating them with an expanded laser beam, passing through the optical window of the cryostat. The grains illuminated by laser radiation were recorded using the video camera through the optical window of the cryostat located at an angle of 90° to the illuminating laser beam. The resulting video images were processed by specially developed computer programs, as a result of which we obtained the coordinates of the grains, as well as their trajectories *r*_*p*_(*t*), their velocity ${v}_{p}$, acceleration ${a}_{p}$ and mean-square displacements < Δ*r*^2^(*t*)) > .

### Data analysis

For visualization, the laser radiation scattered by the grains is recorded by a video camera. The resulting video recording of grains in the structure is processed by the following software algorithm. First, static noise in the video image is detected and removed, and low- and high-frequency spatial noise is filtered. Next, the search for local maxima in the filtered image is performed. For each maximum, the weighted average coordinate and the integrated brightness of circles considered grains are determined, and then images with brightness values that are too low are deleted. Next, the search for grains on all frames of the video (to restore their trajectory) is carried out using the maximum likelihood method. Likelihood is calculated from the relative displacements of a grain from frame to frame and its brightness, taking into account the behavior of neighboring grains. Finally, the heuristic algorithm processes special cases (intersections and discontinuities of trajectories) and determines the final set of grain trajectories.

The analysis of the obtained video data makes it possible to determine the coordinates of single grains at each moment of time, while the analysis of the displacements of grains for the interframe interval gives the speed ${v}_{p}$ of their motion. Based on the data about the velocities of all grains of a dusty system at each moment of time, it is possible to obtain the velocity distribution of grains and their average kinetic energy of grain motion ${E}_{k}={m}_{p}{v}_{p}^{2}/2$.

## Results and discussions

### Grain levitation in the magnetic trap

**Figure 2:**
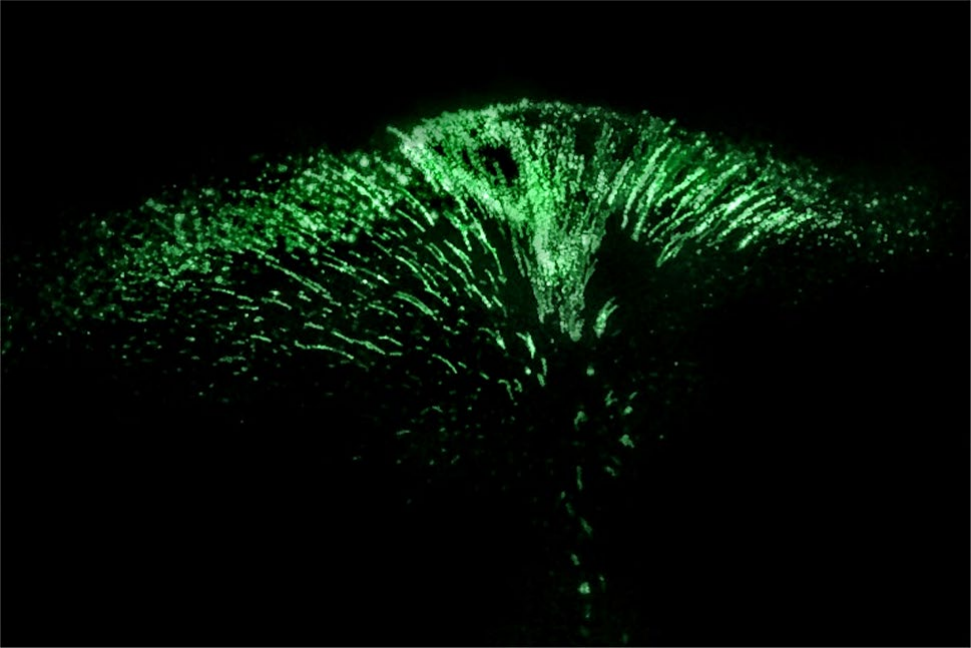
Video frame of a cloud of YBa_2_Cu_3_O_7_ grains levitating in superfluid helium. The superconducting ceramic grains are illuminated by laser radiation with a power density $\dot{{Q}_{p}}$= 1.9 W/cm^2^ (*T*_*He*_ = 1.87 K). The structure is illuminated from the right.

In our experiments, the magnitude of the electric potential *φ*_*p*_ applied to the magnets was on the order of 20 V. As a result of the contact of the superconducting ceramic grains with the magnets, grains gained a charge *Z*_*p*_, the value of which can be estimated as *Z*_*p*_ = $4\pi {\varepsilon }_{o}{\varphi }_{p}{(d}_{p}/2)$, where ${\varepsilon }_{o}$= 8.85·10^–12^ F/m is the dielectric constant. For grains with an average diameter of *d*_*p*_≈40 μm, the value of the charge *Z*_*p*_ does not exceed (${Z}_{p}\lesssim$ 2.7·10^5^ *e*), where *e* is the electron charge. As a result, the formation of a complex spatial colloidal structure by grains confined in a magnetic trap in the form of a "jelly fish" was observed, the "core" of which was rather loose, and the upper part ("umbrella") consisted of a cloud of space-oriented chains and single grains, illuminated by laser radiation of various powers *I*_*L*_ = 0.1–1.0 W (see Fig. 2 and Supplementary InformationVideo file “V1.avi”). The characteristic vertical and transverse dimensions of the colloidal structure were approximately 8 and 20 mm, respectively (the cross-sectional area *S*_*str*_ of the structure was approximately 1.6 cm^2^). Such a system of oriented chains is typical for nematic liquid crystals^26^. The number of grains in the chains varied from 5–7 to 15–20, and the average distance between grains in the chains was in the range of 40–70 microns.

The power density of laser radiation ${\dot{Q}}_{L}$=${I}_{L}/{S}_{L}$ (where *S*_*L*_ is the cross-sectional area of the laser beam) on the grain surface varied within ${\dot{Q}}_{L}$≈ 0.37–2.7 W/cm^2^. The power of heat released on one grain could be on the order of *I*_*L*_((*πd*_p_^2^/4)/*S*_*L*_) ≈3 - 24 μW for grains with an average diameter of *d*_*p*_*≈*40 μm, provided that the absorption coefficient of radiation of the grain material in the visible spectrum is 0.7. In this case, the power density of heat release $\dot{{Q}_{p}}$ on the surface of the grains is in the range 0.26–1.9 W/cm^2^. The power of local heat release depends on the size of the grain and is proportional to its area; therefore, in experiments^27–29^, it was 3–4 orders of magnitude lower.

The formation of chains is influenced by both Coulomb forces (the grains are charged) and magnetic forces (grains of superconducting ceramics can be considered equally oriented magnetic dipoles). At this point, the Coulomb forces *F*_*e*_ are inversely proportional to *r*^2^, and the forces of interaction of magnetic dipoles *F*_*m*_ are inversely proportional to *r*^4^^30^:

1 $${F}_{e}=\frac{1}{4\pi {\varepsilon }_{o}}\frac{{Z}_{p}^{2}}{{r}^{2}}$$

and

2 $${F}_{m}=\frac{3{\mu }_{o}{p}_{m}^{2}}{2\pi {r}^{4}}$$

Here, ${p}_{m}={(\chi }_{p}{V}_{p}/{\mu }_{o}){B}_{m}$ induced magnetic moment of a grain in a magnetic field with induction ${B}_{m}$, ${\mu }_{o}$ =1.26·10^–6^ H/m vacuum permeability, ${\chi }_{p}$ magnetic susceptibility of grain material, and ${V}_{p}$ grain volume.

Estimates of these forces for the experimental conditions at *φ*_*p*_ = 20 V, *r≈*${d}_{p}$, ${\chi }_{p}\approx$ 0.5^31^, ${B}_{m}$=(10–20)·10^–3^ T yield *Z*_*p*_≈2.7·10^5^ *e*, ${p}_{m}$=(1.3–2.6)·10^–10^A·m^2^, *F*_*e*_≈1.1·10^–8^ N and ${F}_{m}$≈(4.0·10^–9^–1.6·10^–8^) N. Note that the condition *F*_*e*_≈${F}_{m}$∽10^–8^ N, which is needed for the formation of chain structures, is met at *Z*_*p*_≈2.7·10^5^ *e*, ${p}_{m}$=2.6·10^–10^ A·m^2^, *φ*_*p*_ = 20 V and ${B}_{m}$=20·10^–3^ T.

### Dynamic behavior and spatial structures of grains

**Figure 3:**
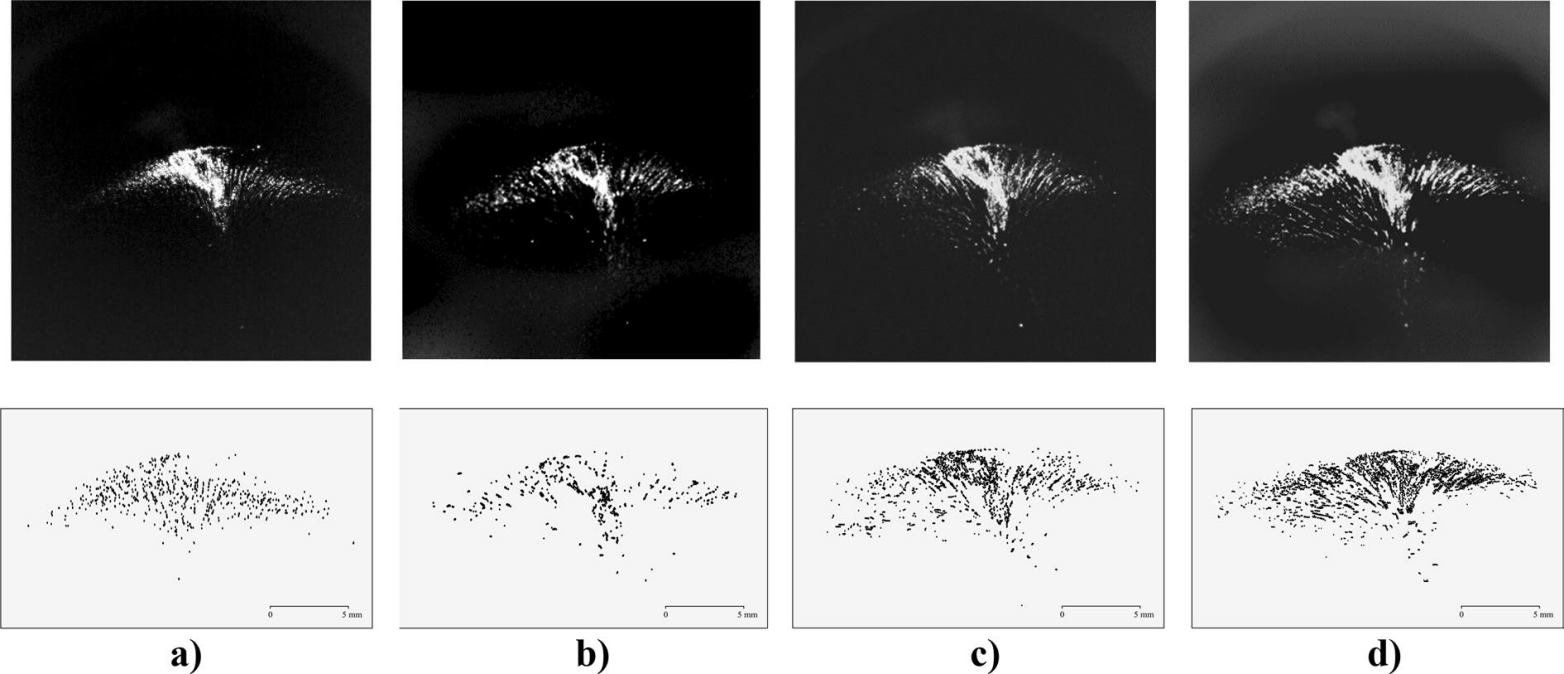
Video frames of a cloud of YBa_2_Cu_3_O_7_ grains levitating in superfluid helium and illuminated by laser radiation of various power densities ${Q}_{p}$**.** Top row: (**a**) $\dot{{Q}_{p}}$ = 0.3 W/cm^2^ (*T*_*He*_ = 1.72 K); (**b**) $\dot{{Q}_{p}}$= 0.7 W/cm^2^ (*T*_*He*_ = 1.87 K); (**c**) $\dot{{Q}_{p}}$= 1.3 W/cm^2^ (*T*_*He*_ = 1.87 K) and (**d**) $\dot{{Q}_{p}}$= 1.9 W/cm^2^ (*T*_*He*_ = 1.87 K). Bottom row: the trajectories of grains motion during 0.1 s. The structure is illuminated from the left.

Table 1 shows the results of an analysis of the dynamic behavior of grains in the structure at various laser radiation powers in superfluid helium at *T*_*He*_ = 1.72 K and 1.87 K, and the corresponding grain structures are shown in Fig. 3 (see also Supplementary InformationVideo file “V2.avi”). When estimating, we used the grain with a diameter *d*_*p *_*≈ *40 μm and mass *m*_*p *_≈ 2.1‧10^–10^ kg, and we analyzed grains in the upper part only (in "umbrella").

**Table 1:** The dynamic behavior of grains in the structure under the action of laser radiation of different powers.

Experiment	1	2	3	4
*T*_*He*_*,* K	1.72	1.87	1.87	1.87
$\dot{{Q}_{p}}$, W/cm^2^ (% from max power density)	0.3 (20%)	0.7 (40%)	1.3 (70%)	1.9 (100%)
Average grain velocity *v*_*p*_ in a laser beam, mm/s (over the ensemble of moving grains)	0.22	0.52	0.96	1.12
Maximum speed of a single grain, mm/s	1.1	1.2	3.8	3.6
Kinetic energy of grains ${E}_{k}={m}_{p}{v}_{p}^{2}/2$ in a laser beam, eV (at *m*_*p*_ = 2.1‧10^–10^ kg)	0.32‧10^2^	1.8‧10^2^	6.1‧10^2^	8.3‧10^2^
Specific entropy loss *ΔS*_*k*_/*N*_*p*_, eV/K	− 0.18‧10^2^	− 0.96‧10^2^	− 3.3‧10^2^	− 4.4‧10^2^

One can see from Table 1 that an increase in the power $\dot{{Q}_{p}}$ of laser radiation of approximately 2.5 times leads to an increase in the grain kinetic energy ${E}_{k}={m}_{p}{v}_{p}^{2}/2$ of approximately 25 times. Note that the grains have an irregular shape (see subplot in Fig. 1a). In addition, we observed random rotation with a characteristic frequency of 50–70 Hz in the experiments.

Thus, there is a mechanism for converting the energy of an external source (laser radiation) into the energy of their kinetic motion, i.e. they are components of an open system, and the motion of the grains themselves can be characterized as the motion of active Brownian grains^7^ with strong Coulomb and magnetic interactions. It should be pointed out that such motion can be treated as hot Brownian motion, in accordance with the definition introduced in^32^. The distinctive feature of the motion is the mechanism involving the heating of the grains to a temperature higher than the ambient temperature (the grains are hotter than the environment). Such grains differ from passive Brownian grains^33^, in which the mechanism of grain motion is associated with thermal fluctuations in the surrounding fluid, and the diffusion coefficient ${D}_{p}$ is determined by the Einstein formula^34^:

3 $${D}_{p}=\frac{{k}_{B}{T}_{He}}{6\pi ({d}_{p}/2){\eta }_{n}}$$

where ${k}_{B}$—Boltzmann's constant, *T*_*He*_—temperature of the liquid, ${\eta }_{n}$—viscosity of the liquid, *d*_*p*_—grain diameter.

**Figure 4:**
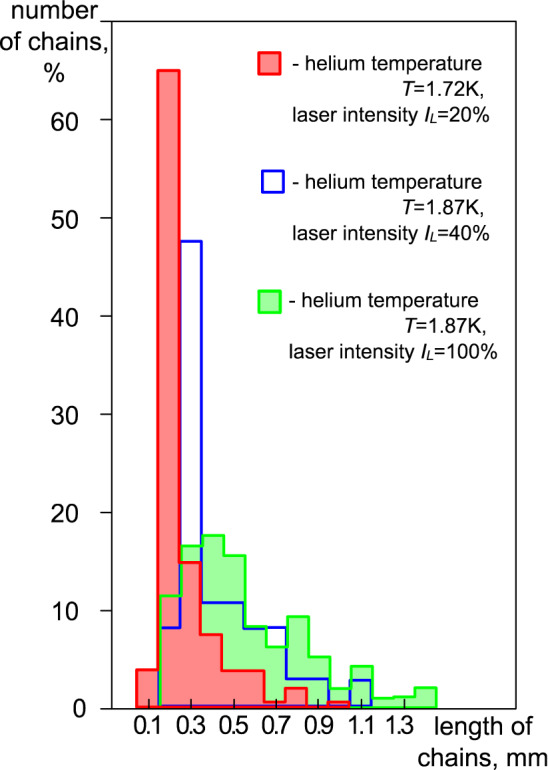
Distribution of chains by lengths in the structure under the action of laser illumination of various power densities $\dot{{Q}_{p}}$: red indicates $\dot{{Q}_{p}}$ = 0.3 W/cm^2^ (*T*_*He*_ = 1.72 K); blue presents $\dot{{Q}_{p}}$ = 0.7 W/cm^2^ (*T*_*He*_ = 1.87 K) and green is for $\dot{{Q}_{p}}$ = 1.9 W/cm^2^ (*T*_*He*_ = 1.87 K).

The results of our observations also show that with an increase in the laser radiation power density $\dot{{Q}_{p}}$, there is not only an increase in the average kinetic energy of grains but also a change in the spatial structures of grains: an increase in the number of chains and their length, while the relative number of single grains decreases (see Table 2 and Fig. 4). Note that with an increase in the value of $\dot{{Q}_{p}}$, the number of observed (visible) grains also increases, which can be explained by the threshold nature of their video recording (a grain can be observed if the scattering of laser radiation on it exceeds a certain threshold of video camera sensitivity).

**Table 2:** The spatial structures of grains under the action of laser radiation of different powers.

Experiment	1	2	3	4
*T*_*He*_, K	1.72	1.87	1.87	1.87
$\dot{{Q}_{p}}$, W/cm^2^ (% from max power density)	0.3 (20%)	0.7 (40%)	1.3 (70%)	1.9 (100%)
Number of observed grains	880	683	1010	1354
Total number of grains in chains	252	212	465	728
Total number of single grains	628	471	545	626
Total number of chains, units	52	38	80	98
Fraction of grains in chains, %	29%	31%	46%	54%
Total length of chains, mm	12	14.4	31.6	49.5
Average chain length, mm	0.230	0.380	0.400	0.500
Maximum chain length, mm	0.73	0.88	1.21	1.35
Number of grains in a chain	12	13	18	20
Maximum chain speed, mm/sec	0.56	1.8	2.4	3.2
Number of grains in a chain	4	5	10	12

### Brownian motion of grains

**Figure 5:**
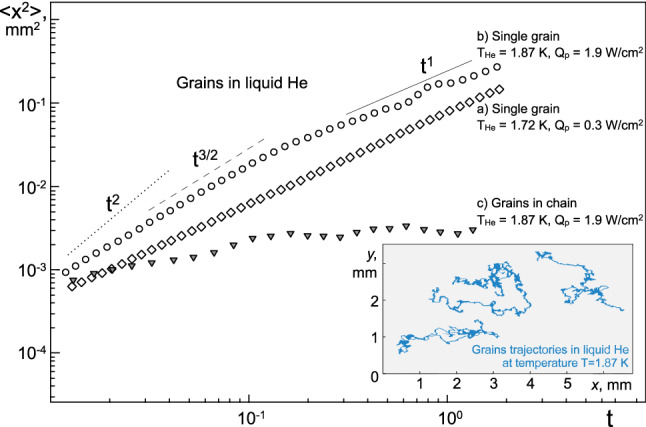
Mean-square displacements of grains in the structure under the action of laser illumination of various power densities $\dot{{Q}_{p}}$: (**a**) single grain at $\dot{{Q}_{p}}$ = 0.3 W/cm^2^ (*T*_*He*_ = 1.72 K); (**b**) single grain at $\dot{{Q}_{p}}$ = 1.9 W/cm^2^ (*T*_*He*_ = 1.87 K) and (**c**) grains within chain at $\dot{{Q}_{p}}$ = 1.9 W/cm^2^ (*T*_*He*_ = 1.87 K). The inset shows the characteristic trajectories of grains.

To analyze the Brownian motion of single grains, as well as grains within the chains, we calculated the experimental time dependences of their mean-square displacement (MSD) < Δ*r*^2^(*t*)) > for various powers of laser illumination (see Fig. 5). The figure plotted in log–log scale shows straight lines corresponding to different modes of Brownian motion MSD ~ *t*^β^^35–37^: ballistic motion (*β* = 2), anomalous diffusion (3/2 < *β* < 2) and normal diffusion (*β* = 1). The characteristic trajectories of motion of single grains in the structure are shown in the inset in Fig. 5. Note that theoretically, the problem of the Brownian motion of a grain in superfluid helium was considered in a number of works^38–42^.

In our experiments, under the action of laser radiation, a change in MSD is observed at characteristic times *τ ≈* 0.02–1.0 s; at longer times (*τ* ≥ 0.7 s). Brownian motion was diffusive with the following diffusion coefficients: ${D}_{p}$≈ 6.0⋅10^–8^ m^2^/s (at *T*_*He*_ = 1.72 K and $\dot{{Q}_{p}}$=0.3 W/cm^2^) and ${D}_{p}$≈ 3.3⋅10^–7^ m^2^/s (at *T*_*He*_ = 1.87 K and $\dot{{Q}_{p}}$=1.9 W/cm^2^). From Einstein's formula (3), the following values of the diffusion coefficients can be obtained: ${D}_{He}$≈ 4.9⋅10^–14^ m^2^/s (*T*_*He*_ = 1.72 K and *η*_*n*_ = 1.29‧10^–6^ Pa‧s^43^) and ${D}_{He}$≈ 5.2⋅10^–14^ m^2^/s (*T*_*He*_ = 1.87 K and *η*_*n*_ = 1.32‧10^–6^ Pa‧s^43^), which are less than those obtained from experiments, by 6–7 orders of magnitude.

For short times (0.02 ≤ *τ* ≤ 0.7 c), grain diffusion is abnormal, < Δ*r*^2^(*t*)) >  ~ *t*^β^, where 1 < *β* < 3/2 (superdiffusion)^1,35–37^.

The experimentally obtained values of the diffusion coefficients agree by order of magnitude with those calculated from formula (3) at ${k}_{B}{T}{\approx {E}_{k}=m}_{p}{v}_{p}^{2}/2$: ${D}_{p}$≈ 1.0⋅10^–8^ m^2^/s (${E}_{k}\approx$ 0.32‧10^2^ eV at *T*_*He*_ = 1.72 K and $\dot{{Q}_{p}}$=0.34 W/cm^2^) and ${D}_{p}$≈ 2.7⋅10^–7^ m^2^/s (${E}_{k}\approx$ 8.3‧10^2^ eV at *T*_*He*_ = 1.87 K and $\dot{{Q}_{p}}$=1.9 W/cm^2^).

For the Brownian motion of grains in a classical liquid (non quantum), the ballistic regime (< Δ*r*^2^(*t*)) >  ~ *t*^2^) takes place at times^44–46^ $\tau \le {m}_{p}/(6\pi ({d}_{p}/2){\eta }_{n})$ ≈ 0.4 s (at *T*_*He*_ = 1.72 K and *T*_*He*_ = 1.87 K), whereas in our case, grains’ motion is not ballistic even at *τ≈* 0.02–0.4 s (see Fig. 5).

Note that the Brownian motion of grains within chains is characterized by the localization of their motion. As a result, the time dependence of the mean-square displacement of grains in the chain has the form of a plateau (see Fig. 5).

The relative contribution of directed grain motion in comparison with random motion at temperature *T*_*He*_ can be characterized by the Peclet number^47^:

4 $$Pe=\frac{{{d}_{p}v}_{p}}{{D}_{He}}$$

At ${D}_{p}$≈ 4.9⋅10^–14^ m^2^/s (*T*_*He*_ = 1.72 K and $\dot{{Q}_{p}}$=0.3 W/cm^2^) and ${D}_{p}$≈ 5.2⋅10^–14^ m^2^/s (*T*_*He*_ = 1.87 K and $\dot{{Q}_{p}}$=1.9 W/cm^2^), Eq. (4) yields *Pe* ~ 1.8‧10^5^ and *Pe* ~ 8.6‧10^5^, correspondingly. Thus, the intensity of the motion of chains and single grains increases with an increase in the power of laser radiation.

We also gradually reduced the intensity of laser beam radiation acting on the structure of grains to zero, then switched on the laser again and increased the power of its radiation within 3 s to ~ 5% of the maximum value, after which we measured grains’ speed. The average velocity was ${v}_{p}$~0.12 mm/s (at *T*_*He*_ = 1.87 K), which corresponds to kinetic energy ${{E}_{k}=m}_{p}{v}_{p}^{2}/2$≈9.5 eV. At helium temperature *T*_*He*_, the kinetic energy of the grains should have a value of ~ ${k}_{B}{T}_{He}$≈1.6⋅10^–4^ eV.

### Evolutionary process of Brownian grains

Thus, the observed systems of active Brownian grains in superfluid helium can be considered as open systems (there is an exchange of energy with the environment), and the ordered structures of grains are the structures far from equilibrium. Such structures are called dissipative; in these structures, the scattering of the energy coming from outside makes possible a stationary ordered structure with an entropy less than the equilibrium one. In dissipative structures, a change (increase) in the flow of entropy into the environment can occur, which results in their self-organization and evolution^1–4^.

Self-organization and evolution of open systems at all levels occurs due to the outflow of entropy into the environment, while the free energy entering a stationary system should exceed the contribution from the production of entropy in the system^1,2^. In our experiments, grains of superconducting ceramics in superfluid helium obtain energy $\dot{{Q}_{p}}$ when from laser radiation with an effective temperature ${T}_{L}$, which can be calculated from the following relation^48^:

5 $$\dot{{Q}_{p}}=\Delta \lambda \left(\frac{2\pi h{c}^{2}}{{\lambda }^{5}}\right)\frac{1}{{e}^{hc/\lambda {k}_{B}{T}_{L}}-1},$$

Therefore, for ${T}_{L}$, we can write:

6 $${T}_{L}=hc/\lambda {k}_{B}{\left[ln\left(1+\Delta \lambda \left(\frac{2\pi h{c}^{2}}{{\lambda }^{5}}\right)\frac{1}{\dot{{Q}_{p}}}\right)\right]}^{-1}$$

Here, $h$—Planck constant, $c$–light speed, $\lambda =$ 532 nm—laser radiation wavelength, and  $\Delta \lambda$—laser radiation line width. Given that $\dot{{Q}_{p}}$ = 0.26–1.9 W/cm^2^ and $\Delta \lambda$ ≈ 1 nm, we find that ${T}_{L}$ is in the range (3.3–4.4)⋅10^3^ К, which is much higher than the temperature of superfluid helium *T*_*He*_.

Due to the thermal balance in the system, the grains transfer exactly the same amount of energy to superfluid helium. Due to the thermodynamic relation $\delta Q=TdS$, the total export of entropy is equal to^1,2^:

7 $$\frac{{dS}_{p}}{dt}={\dot{Q}}_{p}\left[\frac{1}{{T}_{L}}-\frac{1}{{T}_{He}}\right]<0$$

At *T*_*He*_ = 1.87 К and $\dot{{Q}_{p}}$= 0.26–1.9 W/cm^2^ entropy export density $\frac{{dS}_{p}}{dt}$ is in the range

8 $$\frac{{dS}_{p}}{dt}=-\left(0.14-1.02\right)\mathrm{ W}/{\mathrm{K cm}}^{2}$$

In dissipative structures, part of the decrease in entropy ${\Delta S}_{k}$ of the system, as opposed to the equilibrium case, is due to the kinetic energy *E*_*k*_ of moving grains^49^:

9 $${\Delta S}_{k}=-\frac{{E}_{k}}{{T}_{He}}=-\frac{{N}_{p}\left(\frac{{m}_{p}{v}_{p}^{2}}{2}\right)}{{T}_{He}},$$

where *N*_*p*_ is the number of observed grains in the laser beam.

The calculations of the specific (per grain) entropy loss *ΔS*_*k*_/*N*_*p*_ at *T*_*He*_ = 1.87 K, presented in Table 1, show a significant increase in |*ΔS*_*k*_/*N*_*p*_| in the system of grains (by approximately two orders of magnitude) with an increase in the laser radiation intensity from 0.26 to 1.9 W/cm^2^.

Thus, an increase in the power of laser radiation leads to an increase in the kinetic energy of grains and chains, and we observe a growth in the number of ordered and stable autonomous structures (chains) of grains in the levitating cloud as well. Laser radiation also induces the transition of a strongly nonequilibrium stationary system of ceramic grains to a state with a lower entropy (evolution process), i.e. into a more ordered state.

### Grain motion and quantum turbulence

It is a common fact that the motion of grains in superfluid helium can be quite complex^12^. The normal liquid ^4^He (He I), when cooled below *T*_*λ*_ = 2.177 K (λ-point), undergoes a second-order phase transition; the low-temperature phase is known as He II^49^. The physical properties of He II cannot be described by classical physics; it is a quantum liquid exhibiting unusual physical properties such as superfluidity^11^. Phenomenologically, He II is described by a two-fluid model^50^, in which it consists of two components: a viscous normal fluid with a density *ρ*_*n*_, which carries all the entropy, and a non viscous superfluid component with a density *ρ*_*s*_; the total density of He II is *ρ* = *ρ*_*n*_ + *ρ*_*s*_. At *T*_*λ*_ and higher, helium is a normal liquid, while at the limit of zero temperature, there is no normal liquid. For many practical purposes, He II can be considered as consisted only of a superfluid component at temperatures below 1 K.

The superfluid component has a temperature-dependent density *ρ*_*s*_(*T*) and is 100% of the total fluid density *ρ* at absolute zero (*ρ*_*s*_(*T* = 0) = *ρ*). It has no viscosity and no entropy; on the other hand, a normal fluid (with density *ρ*_*n*_(*T* = *T*_*λ*_) = *ρ*) behaves in the same way as a classical fluid.

Under certain conditions, quantum turbulence occurs in He II, and its circulation is limited by quantized vortex lines, each of which has a quantum of circulation κ ≈ 10^−7^ m^2^/s around the core, with a diameter of approximately *ξ*_*o*_ ≈0.1 nm^51,52^. It was first considered in the work of Feynman^53^, while in a thermally induced flow of He II created by an electric heater, it was experimentally studied and theoretically described by Vinen^52,54–57^. In the presence of a heat source, a normal fluid carries entropy away from the source with a velocity ${v}_{n}$, while a superfluid liquid moves to the heat source with a velocity ${v}_{n}$ so that the total mass flow rate is zero, *ρ*_*n*_ ${v}_{n}$+*ρ*_*s*_ ${v}_{s}$= 0^58^. Thus, a thermal counterflow is created, the relative velocity ${v}_{ns}$*=|*${v}_{n}$*|* +*|*${v}_{s}$*|* of which is proportional to the applied heat flow $\dot{Q}$. It should be noted that the interaction of the normal component with quantized vortices generates nonclassical forces of mutual friction^54–57^.

The normal fluid velocity relates to the magnitude of the heat flux $\dot{Q}$ as^12^:

10 $${v}_{n}=\frac{\dot{Q}}{\rho {S}_{n}{T}_{He}} ,$$

and to the relative velocity of the counterflow as^12^:

11 $${v}_{ns}=\frac{\dot{Q}}{{\rho }_{s}{S}_{n}{T}_{He}} ,$$

where *S*_*n*_ is the specific entropy of the normal component.

When the heat flux increases, the counterflow velocity ${v}_{ns}$ also increases, and turbulence can develop in both components of the liquid^58,59^ when the critical value exceeds ${v}_{ns}^{0}$ ~ 2 mm/s^59–61^. The critical value of the heat power density is ${\dot{Q}}_{cr}$ ~ 20 mW/cm^2^^59,60^.

Superfluid turbulence manifests itself in the form of a quantized vortex tangle^12,59,61,62^, the density *L* of which is determined by the relation:

12 $$L= {\gamma }^{2 }{({v}_{ns}-{v}_{ns}^{0})}^{2}={\gamma }^{2 }{\left(\frac{\dot{Q}}{{\rho }_{s}{S}_{n}{T}_{He}}\right)}^{2}$$

where *γ* is a temperature-dependent parameter^62–64^.

When laser radiation acts on the surface of a ceramic grain, it generates a local heat release, which should cause the movement of the normal component from the grain and the superfluid component to the grain. In our experiments at *T*_*He*_ = 1.72 K (*S*_*n*_ = 3.95⋅10^2^ J/(kg⋅K), *ρ* = 1.45⋅10^2^ kg/m^3^, *ρ*_*s*_ = 1.12⋅10^2^ kg/m^3^^43^) and heat release power density $\dot{Q}$=$\dot{{Q}_{p}}$= 0.26–1.9 W/cm^2^, the velocity of the normal component is in the range of 1.7–12.2 cm/s, and the relative counterflow velocity ${v}_{ns}$ is in the range of 2.1–15.8 cm/s. At *T*_*He*_ = 1.87 K (*S*_*n*_ = 6.27‧10^2^ J/(kg K), *ρ* = 1.45⋅10^2^ kg/m^3^, *ρ*_*s*_ = 0.925⋅10^2^ kg/m^3^^43^) and the same power density of heat release, the velocity of the normal component is in the range of 1.5–11.2 cm/s, and the relative counterflow velocity ${v}_{ns}$ is in the range of 2.4–17.5 cm/s. This means that ${v}_{ns}$> > ${{v}_{ns}^{o}}$.

Thus, the action of laser radiation on grains should lead to the formation of quantum vortices near the grain surface. Such a method of the formation of quantum vortices (their generation by grains during laser heating, in which the grains act as “point” heaters) is fundamentally different from the well-known works in which grains act as vortex markers^59,65^.

As a result, a variety of effects associated with quantum turbulence can arise in such a system. For example, when quantum vortices collide with the grain surface and transfer their momentum to the grain^66^, they can be whirled away by the superfluid component moving in the direction of the laser beam to the heated surface of the ceramic grain. The force *F*_*s*_ acting on a grain when interacting with a superfluid component is determined by the relation^12,62^:

13 $${F}_{s}\approx \frac{{\rho }_{s}{\kappa }^{2}}{4\pi } 2{\beta }_{d}{\left(\frac{{d}_{p}/2}{\mathcal{\ell}}\right)}^{2}\mathrm{ln}\frac{{(d}_{p}/2)}{{\xi }_{0}}$$

where *β*_*d*_ is the geometric factor of the order of unity (*β*_*d*_ ≈1), *ℓ*  = *L*^*−*1/2^ is the average distance between vortices and ((*d*_*p*_/2)/*ℓ*)^2^ is a cross section of the interaction of a grain with a system of quantum vortices. When deriving relation (13), it was assumed that (*d*_*p*_/2) >  > *ℓ*^12,62^.

For comparison, we also estimate the force ${F}_{r}$, which can arise during momentum transfer to a grain by the normal component. This is the case for a potential (vortex-free) helium flow when the superfluid component transforms into a normal liquid to carry away heat from the grain surface^67^:

14 $${F}_{r}\approx \frac{\dot{Q}}{{S}_{n}{T}_{He}}\pi {{(d}_{p}/2)}^{2}{v}_{s}={\left(\frac{\dot{Q}}{{S}_{n}{T}_{He}}\right)}^{2}\pi {{(d}_{p}/2)}^{2}\frac{{\rho }_{n}}{{\rho }_{s}\rho }$$

Let us estimate the force ${F}_{n}$ of the normal component acting on a ceramic grain moving with a velocity ${v}_{p}$ at different laser radiation powers using the Stokes law^12^:

15 $${F}_{n}=6\pi ({d}_{p}/2){\eta }_{n}{v}_{p}$$

where *η*_*n*_ viscosity of the normal component of helium.

The energy *E*_*V*_ stored in a vortex tangle near the grain surface can be estimated as

16 $${E}_{V}\sim {d}_{p}^{3}L{E}_{QT}\sim {d}_{p}^{3}L\frac{{\rho }_{s}{\kappa }^{2}}{4\pi }\mathrm{ln}\frac{{(d}_{p}/2)}{{\xi }_{0}}\sim {F}_{s}{d}_{p}\sim {A}_{s}$$

where ${E}_{QT}= \frac{{\rho }_{s}{\kappa }^{2}}{4\pi }\mathrm{ln}\frac{{(d}_{p}/2) }{{\xi }_{0}}$ is the energy per unit length of the quantum vortex and ${A}_{s}\sim { F}_{s}{d}_{p}$ is the characteristic work done by force ${F}_{s}$.

The results of estimates of the averaged forces ${F}_{n}$, ${F}_{s}$ and ${F}_{r}$ for grains in the structure, obtained from experimental data on the velocities of ceramic grains at *T*_*He*_ = 1.72 K (*η*_*n*_ = 1.29‧10^–6^ Pa‧s) and *T*_*He*_ = 1.87 K (*η*_*n*_ = 1.32‧10^–6^ Pa‧s) are presented in Table 3 at different powers of laser radiation. The density of quantum vortices at different powers of laser radiation is estimated by formula (12) at *γ*≈200 s/cm^2^^64^ and *β*_*d*_ ~ 1^12,62^.

**Table 3:** The averaged forces ${F}_{\Sigma }$, ${F}_{s}$, ${F}_{n}$ and ${F}_{r}$ for grains in the structure, obtained from experimental data on the velocities of ceramic grains.

Experiment	1	2	3	4
*T*_*He*_	1.72 K	1.87 K	1.87 K	1.87 K
$\dot{{Q}_{p}}$, W/cm^2^	0.3 (20%)	0.7 (40%)	1.3 (70%)	1.9 (100%)
Quantum vortex density *L*, 10^10^ m^-2^	0.81	1.9	5.8	12.3
Mean distance between vortices ${\mathcal{\ell}}$, μm	11.1	7.3	4.1	2.9
Average grain speed, mm/s	0.22	0.52	0.96	1.12
Average grain acceleration, mm/s^2^ (over the ensemble of moving grains)	21	–	–	320
Kinetic energy of grains ${E}_{k}={m}_{p}{v}_{p}^{2}/2$ in a laser beam, eV (at *m*_*p*_ = 2.1‧10^–10^ kg)	0.32‧10^2^	1.8‧10^2^	6.1‧10^2^	8.3‧10^2^
Diffusion coefficients: ${D}_{p}$, m^2^/s	6.0⋅10^–8^			3.3⋅10^–7^
Resultant force ${F}_{\Sigma }$, 10^–11^ N	0.44	–	–	6.8
Work done by ${F}_{\Sigma }$ :${A}_{\Sigma }\sim {F}_{\Sigma }{d}_{p}$*,* eV	1.1‧10^3^	–	–	1.7‧10^4^
Specific entropy loss, ${\Delta S}_{\Sigma }$/*N*_*p*_, eV/K	0.64‧10^3^	–	–	0.9‧10^4^
Force ${F}_{s}$ , 10^–11^ N	0.7	1.4	4.2	8.9
Work done by ${F}_{s}: {A}_{s}\sim {F}_{s}{d}_{p}\sim {E}_{V}$*,* eV	1.7‧10^3^	3.5‧10^3^	1.0‧10^4^	2.2‧10^4^
Specific entropy loss, *ΔS*_*s*_/*N*_*p*_, eV/K	− 1.0‧10^3^	− 1.87‧10^3^	− 5.6‧10^3^	− 1.2‧10^4^
Force ${F}_{n}$, 10^–11^ N	0.011	0.026	0.048	0.056
Force ${F}_{r}$ , 10^–10^ N	0.65	2.0	6.23	13.1

The table also shows the resulting force ${F}_{\Sigma }$ acting on the grain and estimates of the characteristic work ${A}_{\Sigma }\sim {F}_{\Sigma }{d}_{p}$ and ${A}_{s}\sim {F}_{s}{d}_{p}$ done by forces ${F}_{\Sigma }$ and ${F}_{s}$. The forces ${F}_{\Sigma }={m}_{p}{a}_{p}$ (at *m*_*p*_ = 2.1‧10^–10^ kg) are obtained from experimental data on the accelerations ${a}_{p}$ of grains moving in superfluid helium. Table 3 also presents estimates of the decrease in entropy in the system of grains, corresponding to the work done by the forces ${F}_{\Sigma }$ and ${F}_{s}$^68^.

We can also evaluate a light pressure force ${F}_{ph}$ acting on grain when the grain is illuminated by laser radiation with heat release power density $\dot{{Q}_{p}}$ on the surface of the grains^69^:

17 $${F}_{ph}\approx \frac{2\dot{Q}}{c}\pi {{(d}_{p}/2)}^{2},$$

where *c* is the velocity of light in a vacuum.

In our experiments at $\dot{{Q}_{p}}$ ≈ 2 W/cm^2^, the force ${F}_{ph}$ is approximately 4‧10^–14^ N. This value is less than the forces ${F}_{\Sigma }$, ${F}_{s}$, ${F}_{n}$ and ${F}_{r}$ by 2–4 orders of magnitude. Thus, the light pressure force does not have a significant effect on the dynamics of the grain motion and can be neglected.

The thermophoresis forces associated with the temperature gradient in superfluid helium near the surface of the grain^70^ can be ignored: as follows from^71^, the temperature jump in superfluid helium over grain size *d*_*p *_≈ 40 μm is negligibly small (less than 1 mK).

The results of estimates performed with an accuracy of an order of magnitude (see Table 3) show that ${F}_{\Sigma }\backsim {F}_{s}$ and ${A}_{\Sigma }{ \sim A}_{s} \sim {E}_{V}$≳*E*_*k*_, while the specific entropy *ΔS*_*s*_/*N*_*p*_ is approximately two orders of magnitude higher than the specific entropy *ΔS*_*k*_/*N*_*p*_. This means that specific entropy loss can be characterized not only by the kinetic motion of ceramic grains but also by the formation of quantum vortices near their surface when the grains are heated by laser radiation.

### Quantum effects in Brownian motion, evolution and interaction of grains

The development of quantum turbulence and the formation of quantum vortices near the grain surface, when they are heated by laser radiation, also has a driving effect on the motion of grains in superfluid helium. In this case, the role of thermal fluctuations in the density of the medium can be played by fluctuations in the density of quantum vortices ∆*L*, which can have a value of *∆L* ~ 0.01^72^, which corresponds to energy fluctuations ${\Delta E}_{V}\sim 0.01{E}_{V} \sim 0.01{A}_{\Sigma }$~10^2^ eV (see Table 3). Note that ${\Delta E}_{V}\sim$ *E*_*k*_ by an order of magnitude (see also Table 1), which is in favor of the above mentioned assumption. The characteristic times of the ballistic motion of grains can be estimated as *τ* ~*ℓ* /${v}_{p}$=*L*^−1/2^/${v}_{p}$~10^–3^–10^–2^ s at *ℓ* ~ 1–10 μm and ${v}_{p}$~0.1–1.0 mm/s.

In the experiments, the action of the laser radiation can lead to the nonuniform heating of the grain surface. This is due to inhomogeneous illumination of the surface of single grains when the grain size is much larger than the wavelength of laser radiation (in the approximation of geometric optics)^73^. In addition, the grains have an irregular shape. Thus, their surface is heated unevenly so that the density of quantum vortices varies along the surface (see relation (12)); hence, spontaneous symmetry breaking associated with the space–time change in the density of quantum vortices is observed^74,75^. As a result, the grain gets an uncompensated impulse (see relation (13)), fluctuating in magnitude and direction, and the intensity of Brownian motion of the grain, both translational and rotational, increases.

It should be noted that the grain motion is hot Brownian motion, which, due to spontaneous symmetry breaking, can be treated as active Brownian motion^32^.

Such a mechanism of motion of grains can be considered as an experimental implementation of a quantum Brownian motor in a regime where quantum fluctuation effects become significant for the transport properties^76,77^.

Thus, an increase in the kinetic energy in a system of grains and an increase in the energy of quantum vortices (and thus a decrease in entropy in the system) is due to the mechanism of heat loss from the surface of grains by the normal component of helium, including the formation of quantum vortices (quantum turbulence). In this case, relation (7) can be transformed to the form:

18 $$\frac{{dS}_{p}}{dt}={\dot{Q}}_{p}\left[\frac{1}{{T}_{L}}-\frac{1}{{T}_{He}}\right]\approx -\frac{{\dot{Q}}_{p}}{{T}_{He}}=-{v}_{n}\rho {S}_{n}<0$$

We assume that laser radiation induces a transition (evolution) of a stationary system of ceramic grains far from equilibrium to a more ordered state (which is also a stationary state far from equilibrium), i.e. into a state with less entropy. Note that a negative entropy flux in a cloud of macroscopic grains (which leads to a decrease in entropy in the system) is created by a kind of quantum entropic pump^78^, whose work consists of entropy loss from the system by the normal component of superfluid helium with a speed ${v}_{n}$≲10.0 mm/s, at this $\frac{{dS}_{p}}{dt}$∽10^4^ W/(m^2^K).

Above the λ-point, the thermal conductivity of liquid helium has the value κ∽2.0·10^–2^ W/(m·K), so we can estimate the following:

19 $$\frac{{dS}_{p}}{dt}\approx -\frac{{\dot{Q}}_{p}}{{T}_{He}}=-\kappa \frac{\Delta T}{\Delta r}\frac{1}{{T}_{He}}$$

At $\Delta T$≈ 1 К, $\Delta r$≈*d*_*p*_≈4·10^–5^ m, we obtain $\frac{{dS}_{p}}{dt}$∽ 10^2^ W/(m^2^К), which is much less (by 1.5—2 orders of magnitude) than the above estimate for the conditions of superfluid helium.

**Figure 6:**
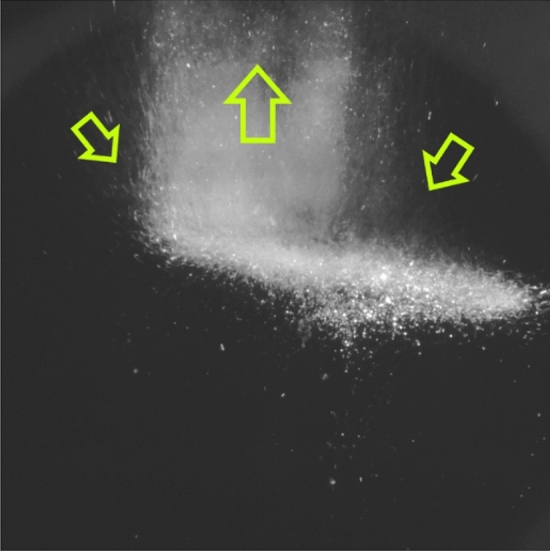
Processes in a structure exposed to laser radiation at *T* = 2.17 K. Video frame of a cloud of superconducting ceramic grains levitating in superfluid helium, carried away by an ascending convective flow from the overheated zone. The direction of motion of the grains is shown by arrows. The structure is illuminated from the left.

When crossing the λ-point, the character of grain motion changes qualitatively: grains are carried out by the ascending convective flow from the overheated zone (see Fig. 6 and Supplementary InformationVideo file “V3.avi”). The grain velocity in this case is on the order of 1 m/s, since we did not detect grains upstream that were sequentially recorded for at least 2 frames of the video recording (at a frame rate of 200 frames/s). The height of the observation area above the structure is approximately 5 mm, which gives an estimate of the minimum speed of 1 m/s.

In superfluid helium, the density of quantum vortices depends quadratically on the power density of laser radiation. The size of the region in which quantum vortex tangles occur is also proportional to laser radiation. To estimate the characteristic dimension of the region of quantum turbulence, we use the relation^79^:

20 $${v}_{n}4\pi {r}^{2}\rho {S}_{n}=\frac{{I}_{L}}{{T}_{He}}$$

We assume that the threshold power density of thermal radiation for the formation of quantum turbulence on the surface of a grain with a diameter of *d*_*p*_ = 40 μm is ${\dot{Q}}_{cr}$ = 20 mW/cm^2^. Then, an increase in the power density of heat release under the action of laser radiation on the grain surface up to values $\dot{{Q}_{p}}$ = 0.26–1.9 W/cm^2^ increases the size of the *D*_*QT*_ region of quantum turbulence up to values *D*_*QT*_ = *d*_*p*_($\dot{{Q}_{p}}$/${\dot{Q}}_{cr}$)½ ~ (4–10)*d*_*p*_ ≈160–400 μm.

**Figure 7:**
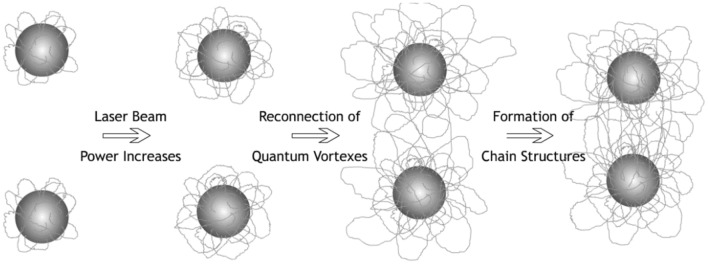
Schematic illustration of the spatial picture of quantum vortices and grains captured by them.

The spatial picture of quantum vortices and grains captured by them is schematically shown in Fig. 7. Note that the charge of the grains in the experiment remained unchanged, and the magnetic moment of the grains depends only on the spatial position (the magnetic field is inhomogeneous, see Fig. 1b).

Such a density growth of quantum vortices increases the effective cross section for the interaction of two grains with the value $\sim {D}_{QT}^{2}\sim {d}_{p}^{2}(\dot{{Q}_{p}}/{\dot{Q}}_{cr})$ due to the overlap of vortices’ tangle and their reconnection (see Fig. 7) and thus increases the probability of capture of the nearest grains and their subsequent confinement into chain structures. The characteristic volume of grain capture per unit time is $\sim {D}_{QT}^{2}{v}_{p}\sim {d}_{p}^{2}(\dot{{Q}_{p}}/{\dot{Q}}_{cr}){v}_{p}$ and can increase by approximately 30 times with a change in the heat release of laser power $\dot{{Q}_{p}}$ from 0.26 W/cm^2^ to 1.9 W/cm^2^. Since the energy of a quantum vortex depends on the length of the chain and it is energetically favorable for it to decrease its length^12^, a tension force arises, which can pull grains or chains together, resulting in an increase in the number of chains and their length^62^. This is confirmed by the estimates given in Table 3, which show that ${A}_{s}\backsim {E}_{V}$≳‧*E*_*k*_ .

**Figure 8:**
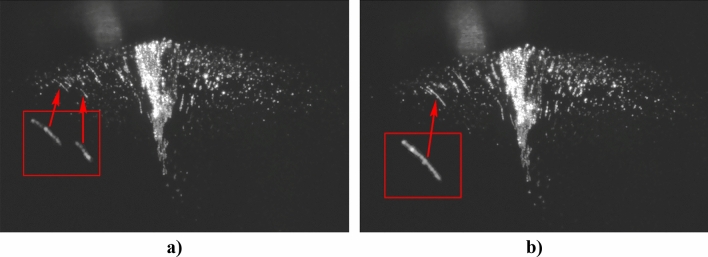
Video frames of the chain formation as a result of a collision of two moving fragments at speeds *v*_*p*_ ≈ 2–3 mm/s. (**a**) before the collision; (**b**) after the collision. The structure is illuminated from the left.

Let us estimate the characteristic binding energy *E*_*b*_ of grains in a chain. For this, we use the results of experimental observation of the formation of a chain due to the collision of a chain moving with a velocity ${v}_{p}$ = 2–3 mm/s with a motionless chain confined in the structure (see Fig. 8 and Supplementary InformationVideo file “V4.avi”):

21 $${E}_{b}\gtrsim {{E}_{k}\sim }\frac{{m}_{p}{v}_{p}^{2}}{2}\sim {F}_{e} {d}_{p}\sim {{F}_{m}d}_{p}$$

Here, ${E}_{k}$—kinetic energy of the grain in the chain. For ${v}_{p}$ =3 mm/s and ${p}_{m}$=1.3·10^–10^ A·m^2^ one can see that *F*_*e*_ ~ ${F}_{m}$~2.4‧10^–11^ N at intergrain distance *r*_*min*_ ~ 200 μm. Note that the obtained values *F*_*e*_ ~ ${F}_{m}$~${F}_{s}$, that is why tension forces associated with quantum vortices can make a significant contribution to the trapping of grains during the formation of chains.

Note that an estimate of the characteristic binding energy *E*_*b*_ of grains in the chain was also calculated using data from experimental observation of the transition of helium from the normal to the superfluid state. In this case, before the formation of chains, the grains can move at speeds up to 5–6 cm/s. It corresponds to the value ${E}_{b}$∽4.7‧10^6^ eV, which is in good agreement (by the order of magnitude) with the energy of grain trapping in the chain by the forces *F*_*e*_≈${F}_{m}$∽10^–8^ N, which in turn is comparable with work done by these forces ${A}_{e}\sim {F}_{e}{d}_{p}\sim {A}_{m}\sim {F}_{m}{d}_{p}$~2.5‧10^6^ eV.

## Conclusions

We present the results of a study of the Brownian motion of grains of superconducting ceramics with sizes up to 60 μm and electric charges up to 10^5^ *e* levitating in a static magnetic trap in superfluid helium (cryogenic colloid) at temperatures of 1.7–2.18 K under the action of laser radiation with a power density of up to ~ 2 W/cm^2^. It is revealed that growth of the power density of laser radiation increases the kinetic energy of the motion of grains and their diffusion coefficient by 6–7 orders of magnitude compared to equilibrium values at temperatures of superfluid helium. It is shown that the motion of grains can be regarded as a motion of active Brownian grains with strong Coulomb and magnetic interactions.

The active Brownian motion of grains in superfluid helium under experimental conditions (at temperatures below the λ-point) is associated with the interaction of the normal and superfluid components of He with the grain surface and with each other due to the appearance of a quantum vortex tangle when the grain surface is heated by laser radiation. The nature of the macroscopic motion of grains changes drastically when helium transits into the normal state, and the grains are carried away by the convection flow of heated helium into the field of gravity. Thus, this active Brownian macroscopic motion is associated with quantum turbulence.

It is also shown that an increase in the laser radiation, when it acts on a cloud of grains, induces the self-organization and evolution of the grain’s system, with the transitions of the system to a more complex state with a lower entropy, while the negative flux of entropy in the system is created due to the quantum mechanism of exceedingly high entropy loss in superfluid helium, associated with the transfer of heat by the normal component of superfluid helium, with the formation of quantum vortices. The most unexpected outcome here is the increase in the number and length of grain chains with the increase in the power density of laser radiation.

Thus, grain structures in our experiments in superfluid helium can be considered dissipative structures, that is, stationary systems far from equilibrium, in which active Brownian motion, as well as the evolution of structures, were driven by quantum effects.

## Supplementary Information

Supplementary Information 1.

Supplementary Video 1.

Supplementary Video 2.

Supplementary Video 3.

Supplementary Video 4.
