# Supplementary material for: Experimental evolution of active Brownian grains driven by quantum effects in superfluid helium
Source: Sci Rep. 2022 Apr 12;12:6085. doi: 10.1038/s41598-022-09523-z (PMC9005707; doi:10.1038/s41598-022-09523-z)
Supplement: Supplementary file 1 — Supplementary Information 1. [file 41598_2022_9523_MOESM1_ESM.pdf]

SupplementaryVideo files
(Titles and short legends)

V1.mp4 –Grain Structure in Superfluid Helium Levitating in a Magnetic Field.
Cloud of $YBa_2Cu_3O_7$ superconducting ceramic grains levitating in superfluid helium when illuminated by laser radiation with a power density Q_p= 1.9 W/cm^2 (T_{He}=1.87 K)

V2.mp4 – Evolution of Grain Structure in Superfluid Helium Levitating in a Magnetic Field under the Action of Laser Radiation.
Cloud of grains of superconducting ceramics $YBa_2Cu_3O_7$ levitating in superfluid helium when illuminated by laser radiation of various power densities Q_p= 0.34 W/cm^2(T_{He}=1.72 K); Q_p = 1.9 W/cm^2(T_{He}=1.87 K)

V3.mp4 – Processes in a Grain Structure Exposed by Laser Radiation at T= 2.17 K.
Processes in a structure exposed by laser radiation at T= 2.17 K: Cloud of superconducting ceramic grains levitating in superfluid helium, carried away by an ascending convective flow from the overheated zone

V4.mp4 –Chain Formation in Superfluid Helium.
The chain formation as a result of a collision two moving fragments at the speeds $v_p \approx$ 2-3 mm/s
